# Post-partum Fever of Unknown Origin: An Inaugural Flare of Severe Lupus With Multisystemic Involvement and Hemophagocytic Syndrome

**DOI:** 10.7759/cureus.33348

**Published:** 2023-01-04

**Authors:** Marco Fernandes, Pedro Ferreira, Ana Lynce, Maria João Correia, Ana Margarida Ribeiro

**Affiliations:** 1 Internal Medicine, Hospital São Francisco Xavier, Lisbon, PRT

**Keywords:** cyclophosphamide pulse, lupus flare, hemophagocytic lymphohistiocytosis (hlh), protein-losing enteropathy, systemic lupus erythromatosus

## Abstract

Systemic lupus erythematosus (SLE) is an autoimmune disease that can affect almost every organ. Lupus protein-losing enteropathy (PLE) is one of the rarest manifestations of gastrointestinal involvement. Lupus flare as initial presentation is rare and the disease can act as a trigger to other pathologic immune syndromes like Hemophagocytic Lymphohistiocytosis (HLH), although this association is rare.

We report the case of a previously healthy African 39-year-old female patient, with a recent history of cesarean section. Admitted to the Emergency Department (ED) with diffuse abdominal pain and fever, having completed a cycle of antibiotic therapy for initially suspected endometritis. The clinical picture progressed with sustained high fever, new-onset lymphadenopathies, systemic rash, acute pulmonary edema and seizures. Laboratory findings included hyperferritinemia, hypertriglyceridemia, proteinuria and hypoalbuminemia. The auto-immune panel was positive for antinuclear antibodies (ANA), anti-dsDNA, anti-SSA and anti-SSB, anti-PL7, anti-RNP, anti-U1-SnRNP, and anti-Pm-Scl75. She also presented hypocomplementemia. An inaugural flare of SLE with multisystemic involvement and concomitant secondary Hemophagocytic Syndrome was considered and therapy with methylprednisolone pulses, Anakinra and Cyclophosphamide was started. By the end of the first cycle of cyclophosphamide, the patient presented clinical worsening with abdominal pain recrudescence and profuse diarrhea. After the exclusion of an infectious process, a Lupus PLE was assumed and Cyclophosphamide protocol was resumed, with sustained clinical improvement after the induction protocol.

Despite initially suspected gynecological infection, the clinical progression with multisystemic involvement together with the auto-immune panel made the diagnosis of SLE possible, with other laboratory findings raising the suspicion of HLH. This case represents a rare report of severe SLE with multiple organ involvement accompanied by HLH. Gastrointestinal involvement with PLE added rarity and morbidity to the clinical picture. The case reinforces the idea that when organ dysfunction is due to a severe autoimmune response, supportive treatment can be lifesaving until immunosuppressive drugs reach their full effect.

## Introduction

Systemic lupus erythematosus (SLE) is an autoimmune disease that encompasses a broad spectrum of symptoms and signs with the ability to affect almost every organic system [1-4]. It is marked by periods of remission with no symptoms that alternate with flares of active disease. A severe flare as the initial presentation is rare [1]. There are several risk factors for disease flares including demographic characteristics (African American race, male gender, onset before 25 years), major organ disease (severe cytopenia, neurolupus, nephritis, vasculitis) and immunological activity (low serum C3/C4, high anti-dsDNA), with only 20% of patients with SLE experiencing at least one severe flare during the natural course of the disease [1-3].

Hemophagocytic lymphohistiocytosis (HLH) represents a rare and life-threatening condition in which a pro-inflammatory state leads to uncontrolled macrophage and T-cell activation with widespread hemophagocytosis, culminating in multisystemic dysfunction [5,6]. The association between SLE and Hemophagocytic Syndrome is rare and represents a challenging situation with a reserved prognosis [7].

Gastrointestinal involvement is not as common as other systems, but the most commonly reported manifestations are nausea, vomiting, dysphagia, abdominal pain, constipation, diarrhea, and hemorrhage [4,7,8]. Lupus protein-losing enteropathy (PLE) is an even rarer manifestation of SLE that is characterized by anasarca and severe hypoalbuminemia due to protein loss by the gastrointestinal tract, and diarrhea is present in about 50% of the cases of PLE [4,8-10].

We present the case of a patient with SLE and multisystemic involvement (cutaneous, serous, neurologic, pulmonary, renal, hematologic and gastrointestinal), along with Hemophagocytic Syndrome presenting as an inaugural severe flare and evolving with PLE.

## Case presentation

**Figure 1:**
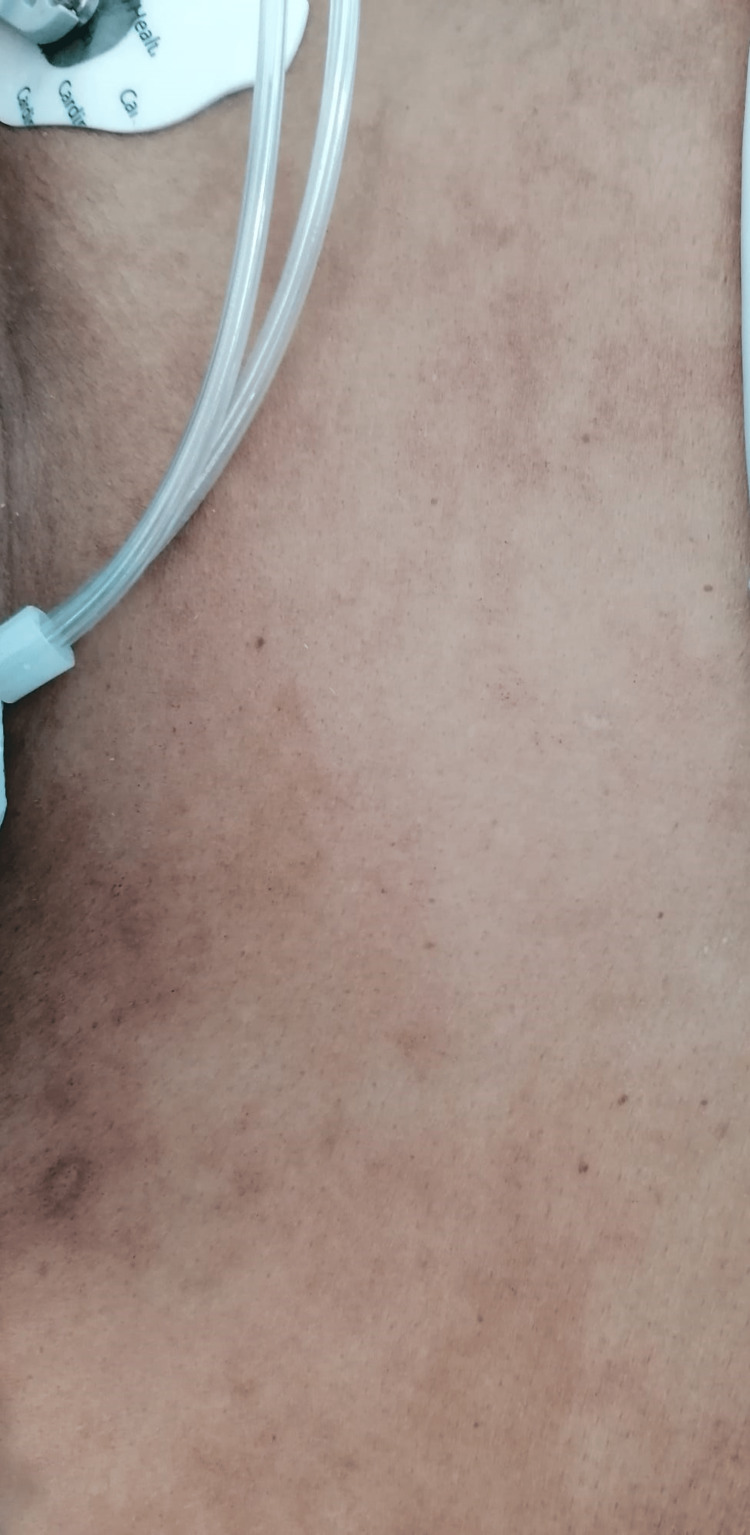
Generalized rash presented by the patient (thoracoabdominal area)

We report the case of a previously healthy African 39-year-old female patient, with a recent history of cesarean section without complications, being discharged after three days of hospitalization. It was her first pregnancy, being submitted to cesarean section due to failure to progress in labor. The newborn was apparently healthy. Approximately fourteen days later, she was admitted to the Emergency Department with diffuse abdominal pain and fever (38º Celsius). Endocervical ultrasound showed the presence of uterine fluid which was drained and sent for microbiologic analysis. Empirical antibiotic therapy was started with cefazolin and the patient was admitted to the Obstetrics and Gynecology Department with the diagnosis of endometritis. Cultural tests isolated Prevotella timonensis and antibiotic therapy was switched to ceftriaxone plus clindamycin and gentamicin, with a decrease of acute phase biomarkers. However, the patient continued to present sustained high fever (38-39,5º Celsius) with new-onset painful cervical and axillary lymphadenopathies and a generalized, non-pruritic maculopapular rash (Figure 1), without the involvement of palmar and plantar surfaces of hands and feet. She was transferred to the Internal Medicine Ward for further investigation

**Figure 2:**
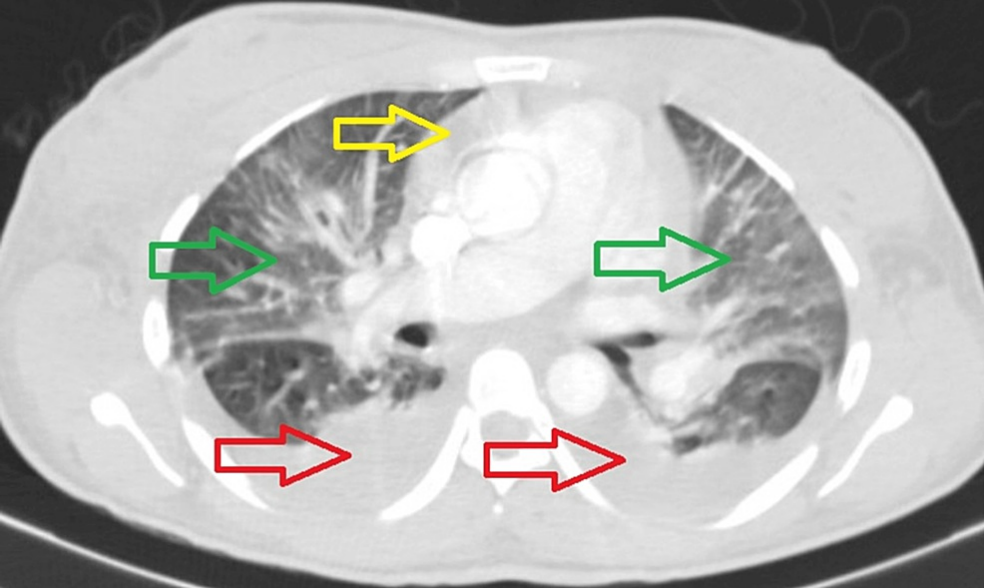
Chest CT scan showing bilateral pleural effusion (red arrows), ground-glass opacities (green arrows), and pericardial effusion (yellow arrow).

She progressed with acute dyspnea and increased work of breathing with impending respiratory failure - the respiratory rate of 30 breaths per minute and pO_2_ of 51 mmHg. Chest x-ray showed diffused bilateral opacities and CT-pulmonary angiography excluded the presence of pulmonary thromboembolism, revealing bilateral pleural effusion, ground-glass opacities dispersed by all pulmonary parenchyma and pericardial effusion (Figure 2).

**Figure 3:**
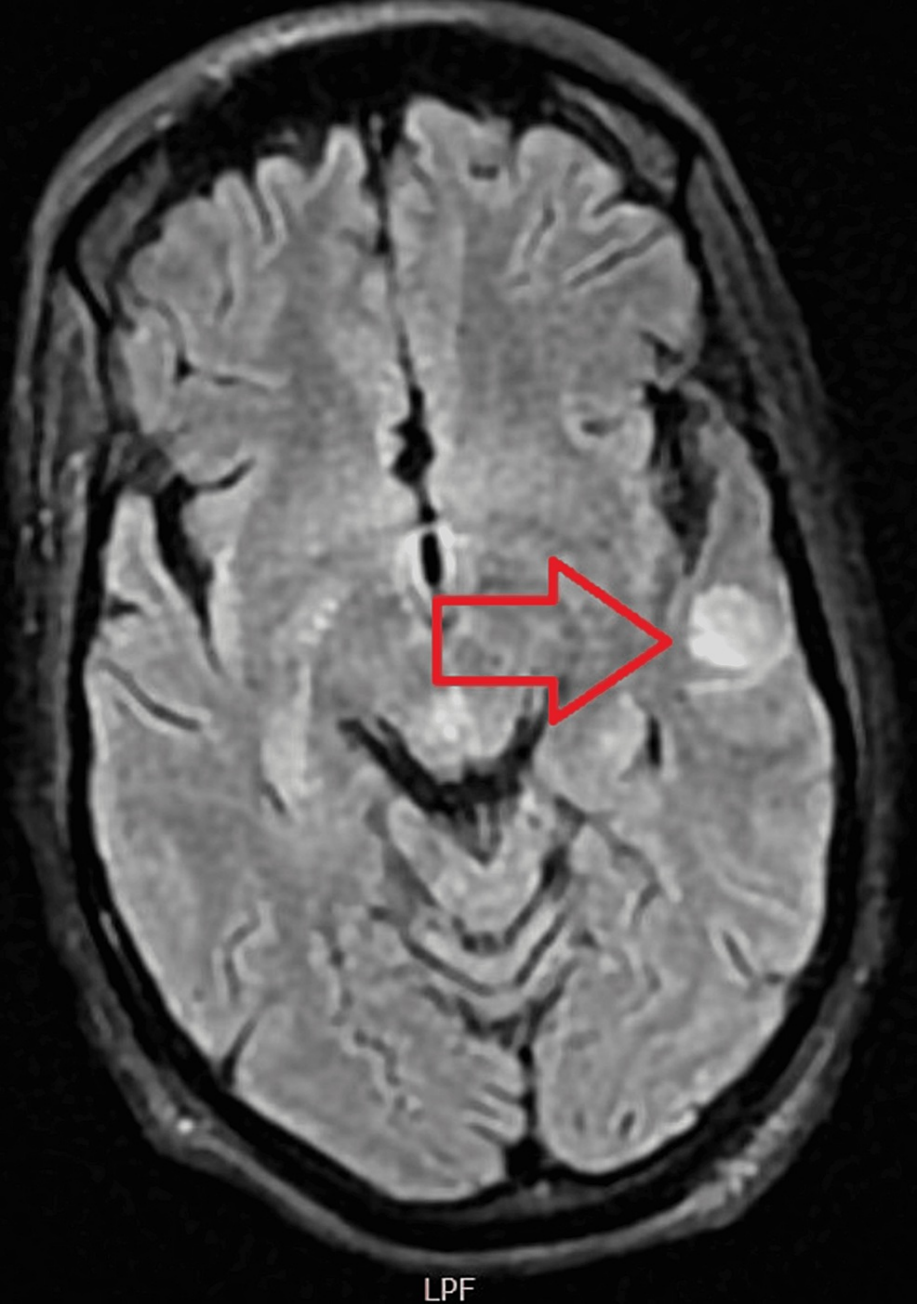
Cranial MRI showing left temporal hyperdensity in T2 sequence (red arrow).

Acute pulmonary edema was assumed, and she showed partial improvement of the respiratory failure on endovenous loop diuretics. Concomitantly, she presented two episodes of tonic-clonic seizures. Head-CT- scan revealed unspecific alterations of density and cranial magnetic resonance imaging (MRI) confirmed the presence of water’s diffusion restriction at cortical areas and revealed the presence of a left temporal hyperdense area at the T2 sequence that could be related to an inflammatory process (Figure 3). A lumbar puncture was performed with no pathological findings after cerebrospinal fluid (CSF) cytological, biochemical and microbiologic examination. Anticonvulsant therapy was started with levetiracetam 1.5g every 12hours with subsequent suppression of abnormal neural activity.

Laboratory findings revealed autoimmune hemolytic anemia, leucopenia, elevated erythrocyte sedimentation rate, hyperferritinemia, hypertriglyceridemia, proteinuria and hypoalbuminemia. The auto-immune panel revealed: positive ANAs (antinuclear antibodies) with a 1:640 titer, hypocomplementemia, and positive anti-dsDNA. Anti-SSA and anti-SSB, anti-PL7, anti-RNP, anti-U1-SnRNP, anti-Pm-Scl75 were also positive. Blood cultures were systematically negative, and IgG for EBV (Epstein-Baar virus) and CMV (Cytomegalovirus) was positive, but IgM was negative. Human Immunodeficiency Virus (HIV), Hepatitis B Virus (HBV), Hepatitis C Virus (HCV), Herpes Simplex Virus (HSV), Human Papillomavirus (HPV) and Human Herpesvirus (HHV)-6,7,8 screening was also negative. Testing for Plasmodium, Toxoplasma, Treponema pallidum, Rickettsia spp and Mycobacterium tuberculosis and spp was negative as well. Laboratory findings and respective evolution are shown in Table 1. Full-body CT-scan revealed hepatomegaly and innumerous cervical and axillary lymph nodes. Cervical node biopsy showed follicular hyperplasia and subcapsular areas with necrosis, compatible with Kikuchi-Fujimoto Disease versus SLE. Medullar bone biopsy showed an increase in megakaryocytic lineage, sins of phagocytic activity, high levels of IL-2 and CD25-CXCL9 positivity. Systemic IL-2 was elevated too (Table 1). Given the proteinuria, the patient was eligible for a kidney biopsy, which could not be performed due to clinical instability.

**Table 1:** Laboratory findings at weeks 1, 2 and 3 after admission MCV - Mean cell volume, MCH - Mean corpuscular hemoglobin, LDH - Lactate dehydrogenase, CMV - Cytomegalovirus, NT - Not tested, ANA- Antinuclear antibodies, ASCA - Anti-Saccharomyces Cerevisiae Antibodies, RBC - Red blood cells, WBC - White blood cells, hpf - high power field.

Laboratory parameters	Week 1 (result)	Week 2 (result)	Week 3 (result)	Normal Range
Hemoglobin (g/dL)	9.4	8.7	6.3	12.0-15.0
MCV (fL)	89	88.8	88.7	80-96.1
Leukocytes (x10^9/L)	4.6	3.5	2.6	4.0 - 10.0
Lynphocytes (%)	11%	10%	3.3%	20-40
Platelets (x 10^9/L)	200	250	222	150-400
C-reactive protein (mg/dL)	6.3	14.5	15.2	<0.5
Sedimentation rate (mm/h)	69	81	90	<35
LDH (U/L)	879	791	1199	135-225
Haptoglobin (mg/dL)	181	11	<10	30-200
Bilirrubin (mg/dL)	0.18	0.24	0.32	<0.9
Ferritin (ng/mL)	656	1822	3542	30-340
Triglycerides (mg/dL)	233	446	443	<150
Albumin (g/dL)	2.3	2.1	1.9	3.5-5.2
Urea, plasma (BUN) (mg/dL)	38	58	72	17-49
Creatinine (mg/dL)	0.62	0.98	1.10	0.5-0.9
Sodium (mmol/L)	138	141	143	136-145
Potassium (mmol/L)	4.1	4.2	4.6	3.5-5.1
Chloride (mmol/L)	102	105	102	98-107
Direct coombs test (positive/negative)	Negative	Positive	Positive	Negative
C3 (mg/dL)	NT	32.4	NT	90-180
C4 (mg/dL)	NT	3.8	NT	10-40
Antinuclear antibodies (positive/negative, titer)	NT	Positive (1:640)	NT	<1:160
anti-dsDNA (UI/mL)	NT	54	NT	30-50
anti-SSA (positive/negative)	NT	Positive	NT	Negative
anti-SSB (positive/negative)	NT	Positive	NT	Negative
anti-PL7 (positive/negative)	NT	Positive	NT	Negative
anti-RNP (positive/negative)	NT	Positive	NT	Negative
anti-U1-SnRNP (positive/negative)	NT	Positive	NT	Negative
anti-Pm-Scl75 (positive/negative)	NT	Positive	NT	Negative
Serum IL-2 (pg/mL)	NT	2855	NT	<2500
CMV viral load (positive/negative)	NT	NT	Negative	Negative
IgG (mg/dL)	NT	1480	NT	600-1500
IgM (mg/dL)	NT	176	NT	50-400
IgA (mg/dL)	NT	61.9	NT	30-300
ASCA (positive/negative)	NT	Negative	NT	Negative
Anti-KLM (positive/negative)	NT	Negative	NT	Negative
Urinary pH	6.2	NT	6.5	4.5 - 8.0
Urinary glucose (mg/dL)	50	NT	80	<130
Urinary ketones (positive/negative)	Negative	NT	Negative	Negative
Urinary nitrites (positive/negative)	Negative	NT	Negative	Negative
Urinary bilirrubin (positive/negative)	Negative	NT	Negative	Negative
Urinary red blood cells (RBC/hpf)	1	NT	0	<2
Urinary white blood cells (WBC/hpf	2	NT	0	<2-5
Proteinuria (positive/negative, mg/dL)	+	+++	1404	<15

Considering an inaugural flare SLE with multisystemic involvement (Systemic Lupus Erythematosus Disease Activity Index of 38 points) and concomitant secondary HLH (Hscore of 216 points), pulses of methylprednisolone were started (1g/day for three days). Anakinra (200mg twice/daily) and Cyclophosphamide according to the Eurolupus protocol were also started. By the end of the first cycle of Cyclophosphamide, she presented a new episode of acute pulmonary edema, delirium, fever recurrence, abdominal pain recrudescence, profuse diarrhea without blood or mucus, the new elevation of acute phase biomarkers and hemolytic anemia worsening (Table 1). Blood cultures, coprocultures, and clostridium screening were all negative as well as cytomegalovirus viral load. Broad-spectrum antibiotherapy with ceftazidime/avibactam was started and, despite negative CMV viral load, ganciclovir was also initiated as well as antifungal therapy with fluconazole. Despite broad antimicrobial therapy and blood transfusion support the patient maintained clinical worsening, evolving with shock and requiring transitory vasopressor, thereby the second cycle of cyclophosphamide was postponed, and she was admitted to the Intensive Care Unit (ICU). Abdominal CT revealed small and large intestine dilatation, vascular engorgement and mesenteric densification which were compatible with inflammatory/ infectious enteropathy. Endoscopic studies ruled out gastrointestinal hemorrhage and biopsies only showed mucosal edema; serum immunoglobulins were normal. ASCA and anti-LKM antibodies were also negative (Table 1). Due to anorexia and severe weight loss, parenteral nutrition was initiated. After three days of supportive care in the ICU, the patient had no recurrence of fever or seizure activity. She also presented respiratory failure improvement and hemolytic anemia resolution. However, she continued to present profuse watery diarrhea with severe hypoproteinemia and hypoalbuminemia.

After the exclusion of an infectious process, a Lupus PLE was assumed, and Cyclophosphamide protocol was resumed. As we resumed the induction protocol, the patient evolved with sustained clinical improvement. Parenteral nutrition was suspended, and oral feeding was tolerated. She was discharged by the end of the fourth cycle of Cyclophosphamide, maintaining follow-up as an outpatient.

## Discussion

SLE may affect multiple organs with a broad spectrum of clinical presentations and complications, making it a notorious mimicker of other disease processes [11]. This case represents an important example of this wide spectrum, with multisystem involvement, presenting as fever in the post-partum period and evolving with shock a few weeks later.

Given the initial presentation with the isolation of a microbiologic agent, the most likable diagnosis was in fact a gynecologic infection. However, sustained fever and the appearance of lymphadenopathies with rash led to further investigation. Discussion with the Infectious disease department led to exclusion of some infectious diseases such as HIV infection, toxoplasmosis, tuberculosis, and others referred to in the blood tests above. 

After transference to the Internal Medicine Ward, further investigation detected lung evolvement and pleural and pericardial effusions, raising the first alarm that we might not be facing an infectious process but instead a systemic inflammatory disease. It is well documented that up to 50% of SLE patients will show any kind of pulmonary involvement [12,13], and up to 40% of the patients may present with asymptomatic pericardial effusion [14].

Laboratory findings showed autoimmune hemolytic anemia, one of the main red flags that lead us to investigate an autoimmune disease, although only 10% of SLE patients show signs of autoimmune hemolysis at initial presentation [15]; she also presented leukopenia, positive ANAs, positive anti-dsDNA and low complement. Thus, by this time, the patient already met 9 Systemic Lupus International Collaborating Clinics (SLICC) criteria: Six clinical criteria (cutaneous, serositis, renal, neurologic, hemolytic anemia, and leukopenia) and 3 immunological criteria (positive ANA, positive anti-dsDNA and low complement). Central nervous system involvement evidenced by seizures (although no changes were found on the CSF examination), added a new clinical criterion to the diagnosis of a severe LES flare. Although not strictly necessary for the SLE diagnosis, the authors considered the importance of a kidney biopsy to confirm renal involvement, given the proteinuria presented by the patient. Unfortunately, the patient had no clinical stability for this exam to be performed.

According to the 2019 update of the European Alliance of Associations for Rheumatology (EULAR) recommendations for the management of SLE, treatment of SLE-related neurologic disease should include glucocorticoids and immunosuppressive agents, which led this patient to be initially treated with cyclophosphamide, besides glucocorticoids.

It has been well-known that autoimmune diseases can lead to the development of HLH, also known as macrophage activation syndrome (MAS) [11]. However, as said before, the association between SLE and HLH is rare with an estimated prevalence between 0.9%-4.6% [16]. The severity of the presentation and laboratory findings like hyperferritinemia raised the concern of an associated macrophage activation syndrome with a Hscore conferring a high probability of this syndrome. Given the severity of this syndrome with high mortality reports, aggressive treatment is advised. Several studies have shown the efficacy of Anakinra in HLH, especially when it’s secondary to rheumatologic disorders [17-19]. The patient’s treatment included a multimodal approach using the EUROLUPUS protocol and adjunctive therapy for HLH with anakinra.

Despite aggressive treatment, the patient showed clinical deterioration and new organ involvement with gastrointestinal symptoms. Given the prolonged hospital stay and severe immunosuppression caused by SLE treatment, an infectious disease had to be excluded and antimicrobial therapy was started. However, the patient continued to worsen evolving with shock and requiring transitory vasopressor. Given the low probability of an infectious process, the authors assumed that the cardiovascular dysfunction and shock presented by the patient were explained by the HLH inflammatory state and severe hypovolemia due to profuse diarrhea. Parenteral nutrition was initiated as a supportive treatment given the anorexia, severe weight loss, and hypoproteinemia presented by the patient in the context of Lupus PLE. This allowed for compensation of the patient's nutritional needs until clinical improvement was achieved. As previously said, PLE is a rare manifestation of gastrointestinal involvement of SLE. In fact, a systemic review suggested that Lupus PLE should be considered in patients with edema and hypoalbuminemia of unknown origin [20].

## Conclusions

This case represents a rare report of severe SLE with multiple organ involvement, accompanied by HLH. It represented a challenging diagnosis from the beginning and throughout hospitalization, with clinical deterioration despite the correct diagnosis and aggressive treatment. Neurological compromise and concomitant HLH made it a severe presentation and gastrointestinal involvement with PLE added rarity and morbidity to the clinical picture. In the presented case, timely diagnosis and high clinical suspicion allowed direct adequate treatment, although supportive treatment was lifesaving until immunosuppressive drugs reached their full effect.
